# Upregulation of IBSP Expression Predicts Poor Prognosis in Patients With Esophageal Squamous Cell Carcinoma

**DOI:** 10.3389/fonc.2019.01117

**Published:** 2019-10-25

**Authors:** Mingyue Wang, Baoxing Liu, Dan Li, Yufeng Wu, Xuan Wu, Shuyue Jiao, Cong Xu, Sheng Yu, Shuai Wang, Jianwei Yang, Yanmei Li, Qiming Wang, Suxia Luo, Hong Tang

**Affiliations:** ^1^Department of Internal Medicine, Affiliated Cancer Hospital of Zhengzhou University, Henan Cancer Hospital, Zhengzhou, China; ^2^Department of Surgery, Affiliated Cancer Hospital of Zhengzhou University, Henan Cancer Hospital, Zhengzhou, China; ^3^Department of Radiology, Affiliated Cancer Hospital of Zhengzhou University, Henan Cancer Hospital, Zhengzhou, China

**Keywords:** ESCC, IBSP, metastasis, invasion, EMT

## Abstract

Esophageal squamous cell carcinoma (ESCC), which is characterized by invasiveness and poor prognosis, is the sixth most common leading cause of cancer-related death worldwide. Despite advances in multimodality therapy, ESCC mortality remains high, and an understanding of the molecular changes that lead to ESCC development and progression remains limited. In the present study, Integrin Binding Sialoprotein (IBSP) upregulation was found in 182 of 269 (67.7%) primary ESCC cells at the mRNA level by quantitative real-time polymerase chain reaction (qRT-PCR). Additionally, IHC staining further demonstrated that IBSP was upregulated in ESCC patients and IBSP protein upregulation was significantly related to the lymph node metastasis (*P* = 0.017), clinicopathologic stage (*P* = 0.001) and poor disease survival *(P* = 0.002). Moreover, functional studies illustrated that the IBSP gene can promote the proliferation and metastasis of ESCC cells. Furthermore, IBSP was found to regulate epithelial-mesenchymal transition (EMT), which promotes tumor cell metastasis. In conclusion, our study suggests that IBSP may be a valuable prognostic marker for ESCC patients.

## Introduction

Esophageal cancer is one of the most common malignancies and has been ranked as the sixth leading cause of cancer death worldwide, especially in the northern part of China (1, 2). Despite advances in multimodality therapy, the prognosis of esophageal cancer remains poor, and the 5-year overall survival is <15% (3, 4). As one of the most common histological forms of esophageal cancer, ESCC is known for its aggression and poor prognosis (5). In addition, previous studies have shown that the occurrence of ESCC is closely related to genetic, environmental, dietary, and other risk factors (6). Similar to other solid tumors, the development of ESCC is an extensive process of oncogene activation and tumor suppressor gene inactivation (7–9). So far, the exact cellular and molecular mechanisms leading to ESCC have not been systematically evaluated.

Genome-wide detection of chromosomal changes was performed, which implicated that the change of 4q is one of the most common genetic changes in ESCC (10, 11), therefore there may be some genes that play important roles in the occurrence and development of ESCC exist in 4q. As a member of the small integrin-binding ligand, N-linked glycoprotein (SIBLING) family, IBSP gene is located in the 4q21.1 region (12–14). IBSP encodes a secreted glycoprotein consisting of 317 amino acids that is mainly expressed in bone tissue (15). This protein binds to calcium and hydroxyapatite via its acidic amino acid clusters and mediates cell attachment through an arginyl-glycyl-aspartic acid (RGD) sequence that recognizes the vitronectin receptor (16–18). Identified as a candidate tumor promoter gene, IBSP exhibits frequent overexpression and upregulation in lung cancer, breast cancer, and prostate cancer (19, 20). However, the relationship between IBSP and ESCC has not been verified. In this study, the expression levels of IBSP mRNA and protein in ESCC samples were detected by qRT-PCR and IHC staining. In addition, the tumor-promoting mechanism of IBSP in ESCC and its clinical significance were also explored.

## Materials and Methods

### ESCC Clinical Samples and Cell Lines

A total of 269 primary ESCC tissues and adjacent normal esophageal epithelial tissues (taken 5 cm away from the tumor edge) were collected at the time of surgical resection at Linzhou People's Hospital (Henan, China). All tumor tissues were histopathologically confirmed as ESCC. Some were quickly placed in vials and stored in liquid nitrogen, while other tumor tissues were routinely fixed with formalin and paraffin embedded. Before operative treatment, none of the patients enrolled in this study received chemotherapy or radiotherapy. The ESCC tissue specimens used in this study were approved by the Committee for the Ethical Review of Research involving Human Subjects at Zhengzhou University. Written informed consent for the original human work that produced the tissue samples was obtained.

The Japanese ESCC cell line KYSE30 was obtained from DSMZ (Braunschweig, Germany), the German Resource Center for Biological Material.

### Quantitative RT-PCR

Total RNA was extracted from cell lines and frozen ESCC tissues by TRIzol (Invitrogen, CA, Carlsbad, USA). According to the manufacturer' instructions, an equal amount of cDNA were synthesized through the application of Advantage RT-for-PCR kit (Clontech). In order to analyze the expression level of corresponding GAPDH and IBSP, qRT-PCR was performed using a SYBR Green PCR Kit (Applied Biosystems) and ABI7500HT Fast Real-Time PCR system (Applied Biosystems). The cDNA was amplified in 30 cycles with the following primers: IBSP forward, 5′-AACAAGGCATAAACGGCACCAGTA-3′ and IBSP reverse, 5′-CGGTAATTGTCCCCACGAGGTT-3′. The GAPDH gene served as an internal control with the following primers: GAPDH forward, 5′-CGGGAAGCTTGTCATCAATGG-3′ and GAPDH reverse, 5′-GGCAGTGATGGCATGGACTG-3′. SDS2.3 software (Applied Biosystems) was employed to analyze relative expression levels. By means of the Ct method, the real-time value for each sample was averaged and compared.

### TMAs and IHC

Tissue microarrays (TMAs) were established with 269 pairs of primary ESCC tumor samples and matched normal esophageal epithelia. The standard streptavidin-biotin-peroxidase complex method was adopted for IHC. Tissue sections with known positive expression were used as the positive control, and phosphate-buffered saline (PBS) was used in place of the primary antibody as a negative control. All of the IHC staining results were independently reviewed by two pathologists. We defined positive cells as cells with the presence of yellow-brown staining in the cytoplasm and the results of IBSP staining were evaluated semi-quantitatively. Compared with the control group, the intensity was measured as follows: 0, negative staining;1, weak staining; 2, moderate staining; 3, strong staining. The positive staining rate of tumor cells were as follows: 0, <1%; 1, 1–10%; 2, 10–50%; 3, 50–75%; 4, >75%. The final score is calculated by adding the percentage and the intensity score, which gives 0 points and 2–7 points. 0–1 is considered a negative number; 2–3 for the weak; 4–5 are moderate; 6–7 is strong. Statistical analysis showed that 0–3 was low expression of IBSP, and 4–7 was over expression of IBSP.

### IBSP ESCC Cell Line Construction With IBSP Upregulation

To verify the function and mechanism in growth assistance by IBSP, PCR amplification, sequencing, and verification were performed on IBSP, which was cloned into the pcDNA3.1 (+) vector (Invitrogen) and transfected into ESCC and KYSE30 cells lacking IBSP expression. A stable IBSP expression clone was selected and resequenced. Blank vector-transfected KYSE30 cells (Vec-30) were used as a control. RT-PCR was employed to detect IBSP expression. GAPDH was used as an internal control, and mRNA and protein expression was quantified by Quantity One software to analyze the relative gray value.

### Tumor Promoter Function of IBSP

To detect the tumor promoter function of the IBSP gene, a focus formation experiment was carried out. For this experiment, 1 × 10^3^ IBSP-30 and Vec-30 cells were seeded in 6-well plates. After 10 days of culture, viable colonies (>50 cells/colony) were counted and stained with Giemsa in repeat three experiments. In addition, an MTT assay was performed to detect the cell growth rates of the IBSP-30 and Vec-30 cells. The cells were seeded in a 96-well plate with a density of 1 × 10^3^ cells/well. Based on the manufacturer's instructions, the cell growth rate was evaluated by employing a cell proliferation MTT kit (Sigma).

### Migration Assays *in vitro*

For the cell migration experiment, IBSP-30 or Vec-30 cells were cultured in 6-well plates until fusion, and then a layer of cells was removed with a sterile needle tip. After culturing for 36 h, photographs were taken under a phase contrast microscope. All independent experiments were performed in triplicate.

### RNA Interfering (RNAi)

According to the manufacturer's instructions, small interfering RNA (siRNA) against IBSP was transfected into IBSP-C2 cells with Lipofectamine 2000 Reagent (Invitrogen). The sequences of siRNAs were as following: siRNA1, GCCACGCTACTTTCTTTATAA and siRNA2,GCATGGCTATGAAGGCTACG (21). 48 h after transfection, the effects of gene silencing were detected by Western blot.

### Western Blotting Analysis

Western blotting was conducted in accordance with standard protocols. The protein-antibody complex was detected by an enhanced chemiluminescence detection system. Quantity One software was used to quantify the protein expression, and GAPDH was analyzed as the relative gray value of the internal control.

### Statistical Analysis

Statistical analysis was conducted with SPSS standard version 21.0. The association between IBSP expression and clinicopathological features was evaluated by the chi-square test or positive probability test. Moreover, disease-specific survival (DSS) was computed from the date of cancer-related death or the date of the last follow-up. The Kaplan-Meier (KM) method was used to generate the survival curve, and the log-rank test was used for statistical analysis. Besides, multivariate analysis of risk factors (IBSP expression, tumor cell differentiation and TNM stage, pN factor) for ESCC was studied by the Cox proportional hazard model. When the *P*-value was <0.05, it was considered statistically significant.

## Results

### Characteristics of the Patients

The study involved 121 males and 148 females with an average age of 59.9 years (40–80 years). According to the 8th edition of the Union for International Cancer Control (UICC) (2017), 17 patients were classified as stage I, 155 as stage II, and 97 as stage III. The follow-up data were obtained from all patients with a median survival of 27 months (3–60 months). The clinicopathological characteristics of the patients are summarized in Table 1.

**Table 1:** Association of the upregulation of IBSP expression with the clinicopathological characteristics of patients with ESCC (*n* = 269).

**Clinicopathological characteristics**	***N***	**IBSP Expression**, ***n*** **(%)**		**χ^**2**^**	***P*-value**
		**Upregulation**	**Normal**		
**Age (years)**					
≤60	148	63, 42.6%	85, 57.4%	0.801	0.384
>60	121	45, 37.2%	76, 62.8%		
**Sex**					
Male	121	53, 43.8%	68, 56.2%	1.221	0.317
Female	148	55, 37.2%	93, 62.8%		
**Tumor cell differentiation**					
Well	32	7, 21.9%	25, 78.1%	6.444	0.040
Moderate	178	72, 40.4%	106, 59.6%		
Poor	59	29, 49.2%	30, 50.8%		
**Lymph node metastasis (*****N*****)**					
N0	149	50, 33.6%	99, 66.4%	6.039	0.017
N1	120	58, 48.3%	62, 51.7%		
**TNM stage**					
I	17	1, 5.9%	16, 94.1%	14.285	0.001
II	155	57, 36.8%	98, 63.2%		
III	97	50, 51.5%	47, 48.5%		
**General classification**					
Medullar type	140	56, 40%	84, 60%	0.098	0.992
Ulcerative type	90	36, 40%	54, 60%		
Sclerotic type	16	7, 43.8%	9, 56.3%		
Mushroom type	23	9, 39.1%	14, 60.9%		

*χ^2^, Chi-square value*.

### Frequent Upregulation of IBSP in ESCCs

**Figure 1:**
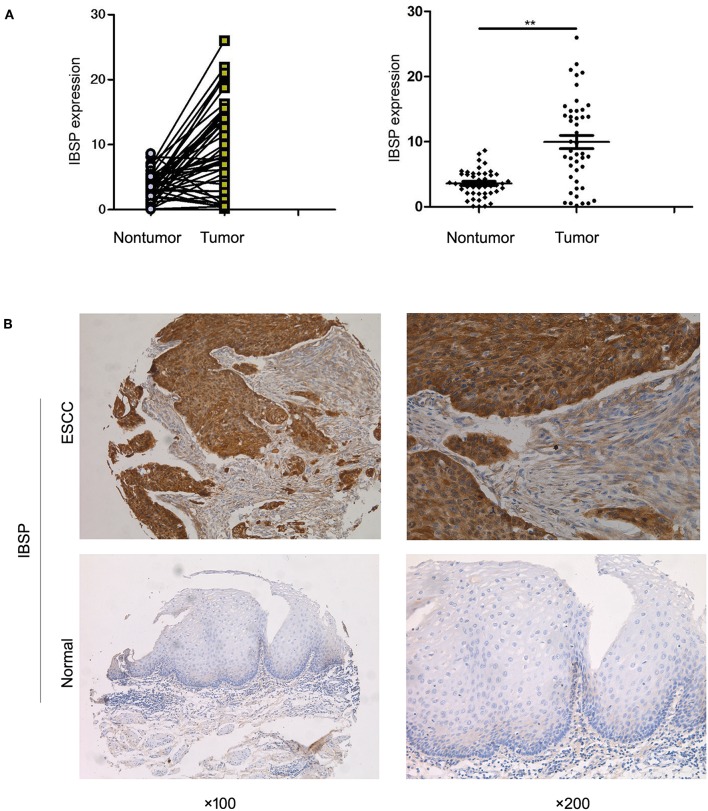
**(A)** Dot-plot graph of the fold change in IBSP in ESCC tissues and their adjacent non-tumor tissues. Scatter plots of fold change in IBSP detected by qRT-PCR in ESCC tissues. **(B)** Representative images of IBSP expression in a pair of ESCC (lower panels) and adjacent normal tissues (upper panels) as detected by immunostaining with the anti-IBSP antibody (brown). The final score of ESCC subgroup is 6 points while that of normal subgroup is 1 point. The slides were counterstained with hematoxylin. Original magnification, ×100 (left), ×200 (right). ^**^P < 0.01.

qRT-PCR was used to evaluate the expression of IBSP at the mRNA level in 269 primary ESCC tumors and their paired normal tissues as well as in the KYSE30 cell line of ESCC. The results showed that IBSP expression was upregulated in 182 of 269 (67.7%) ESCC tumor tissues compared with that in the paired non-tumor tissues (62 of 269,23.0%). Compared with the normal tissues, IBSP were found to express at higher levels in ESCC tissues (*P* < 0.05). The mean level of IBSP expression was markedly higher in ESCC tumor tissues than in their non-tumor counterparts (9.95 vs. 3.59, *P* < 0.001, paired *Student's t-test*; Figure 1A). In addition, we expanded the quantity of samples to investigate the expression of IBSP at the protein level. The IHC results showed that the expression of IBSP was consistent at the protein level in 269 pairs of ESCCs. The data analysis did not include any missing samples, non-representative samples, samples with too few tumor cells, improperly stained samples or other non-informative samples. The expression of IBSP at the protein level was upregulated in ESCC samples (Figure 1B).

### Clinical Significance of IBSP Upregulation in ESCC

**Figure 2:**
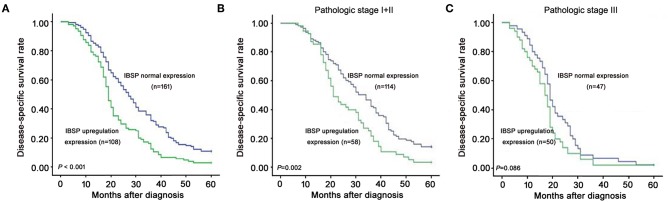
Kaplan-Meier plots for the disease-specific survival rate of ESCC patients. **(A)** Kaplan-Meier plots for the disease-specific survival (DSS) rate of ESCC patients with (*n* = 108, green line) or without (*n* = 161, blue line) IBSP upregulation. Kaplan-Meier plots for the DSS rate in ESCC patients with or without IBSP upregulation subgrouped into pathologic stage I–II **(B)** and pathologic stage III **(C)**.

The relationship between IBSP protein expression and the clinicopathological characteristics of ESCC patients was investigated, and the results showed that the upregulation of IBSP protein was significantly associated with lymph node metastasis (*P* = 0.017) and advanced clinical stage (*P* = 0.001; Table 1) regardless of sex, age, cell differentiation, and general classification. Univariate survival analysis showed that IBSP upregulation, lymph node metastasis status, and advanced tumor metastasis (TNM) were significantly correlated with poor prognosis (*P* < 0.05; Table 2). Moreover, all of these variables with statistical significance in the univariate analysis were further tested by multivariate Cox proportional regression analysis (Table 3). The results showed that IBSP upregulation was an independent risk factor affecting the overall survival of patients (*P* = 0.002), tumor cell differentiation (*P* = 0.001), and TNM stage (*P* = 0.001). KM analysis demonstrated that the DSS time of ESCC patients with upregulated IBSP (median survival time, 19 months) was shorter than that of ESCC patients with normal IBSP expression (median survival time, 23 months) (*P* < 0.001; Figure 2A). We also examined the prognostic value of IBSP expression in ESCC patients with different pathological stages. The results indicated that patients with IBSP upregulation had a significantly shorter DSS rate than those without IBSP upregulation in the I+II subgroup (*n* = 172, *P* = 0.002; Figure 2B), while in the stage III subgroup, there was no significant difference (*n* = 97, *P* = 0.086; Figure 2C).

**Table 2:** Univariate Cox regression analysis of factors possibly influencing disease-specific survival in patients with ESCC.

**Variables**	**HR**	**95% CI**	***P*-value**
IBSP expression	0.573	0.442–0.744	0.000
Age (years)	0.825	0.640–1.063	0.137
Sex	0.872	0.675–1.127	0.295
Tumor cell differentiation	1.557	1.256–1.930	0.000
pN factor	1.968	1.519–2.551	0.000
TNM stage	2.399	1.830–3.147	0.000

**Table 3:** Multivariate Cox regression analysis of factors possibly influencing disease-specific survival in patients with ESCC.

**Variables**	**HR**	**95% CI**	***P*-value**
IBSP expression	0.660	0.505–0.863	0.002
Tumor cell differentiation	1.437	1.158–1.784	0.001
pN factor	1.040	0.637–1.699	0.874
TNM stage	0.442	0.267–0.730	0.001

### IBSP Facilitates ESCC Cell Proliferation and Metastasis

**Figure 3:**
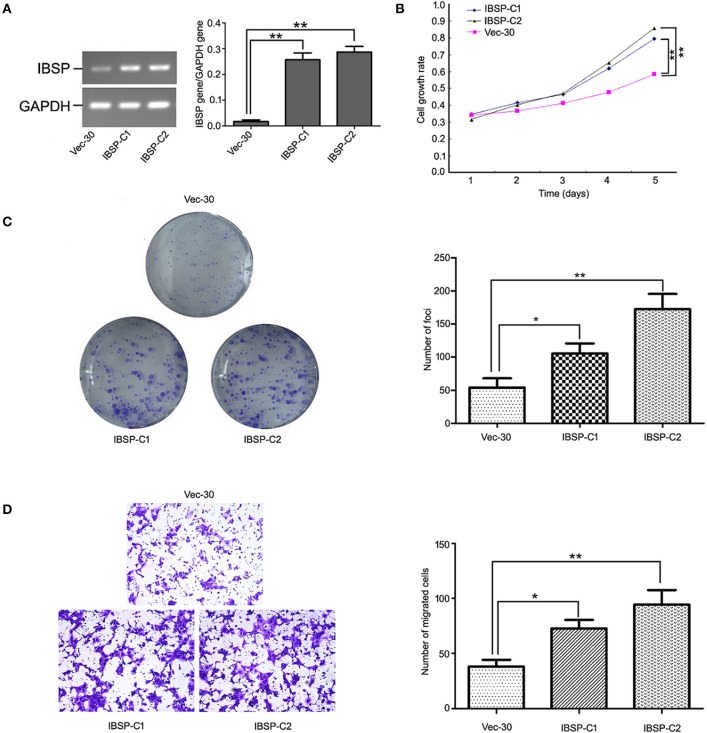
Tumor promoter function of IBSP in ESCC cells. **(A)** Upregulation of IBSP in ESCC. The expression of IBSP in transfected ESCC cells (ibsp-30) was detected by RT-PCR. ^**^P < 0.01. **(B)** The growth curve of IBSP-expressing cells was compared with that of Vec-30 cells by MTT assay. Data points indicate the mean of at least three independent experiments; bars, SD; ^**^P < 0.05. **(C)** Representative auxo-action of IBSP in the foci formation of the culture monolayer and quantitative analyses of the foci quantity are shown. Columns indicate means of at least three independent experiments; bars, SD. ^**^P < 0.05 vs. Vec-30 cells using Student's *t*-test. **(D)** The IBSP and Vec-30 cells that invaded through the Matrigel are shown in representative images. The number of invaded tumor cells is quantified in the histogram. Columns indicate means of triplicate experiments; ^*^P < 0.05, ^**^P < 0.01.

To detect the transfection efficiency of plasmids in IBSP-30 cells, the expression of the IBSP gene in IBSP-30 cells was confirmed by RT-PCR analysis. The ratio of IBSP gene/GAPDH gene expression in Vec-30, IBSP-C1 and IBSP-C2 cells was 0.0098 ± 0.0053, 0.2617 ± 0.0766, and 0.2854 ± 0.1003, respectively (*P* < 0.05; Figure 3A). IBSP expression was higher in IBSP-30 cells than in Vec-30 cells transfected with a blank vector. Additionally, after measuring the OD values for 5 consecutive days, the cell growth assay confirmed that compared with that of Vec-30 cells, the cell proliferation rate of IBSP-30 cells was significantly increased by IBSP (*P* < 0.05; Figure 3B).

The effect of IBSP on tumor development was evaluated by a focus formation assay and cell growth assay. The mean number of colonies formed by IBSP-30 cells was higher than that of Vec-30 cells. Compared with Vec-30 cells, the formation efficiency of IBSP cells was highly improved in the focus formation experiment (*P* < 0.05, Figure 3C). The TMA results demonstrated that the upregulation of IBSP protein expression has a significant relationship with lymph node metastasis, and the effect of IBSP on cell migration was studied by migration experiments. The cell migration assay showed that the number of IBSP-30 cells that migrated was significantly higher than that of Vec-30 cells (Figure 3D). IBSP significantly promoted the migration of ESCC cells compared with that of Vec-30 cells.

### Silencing IBSP Expression by siRNA

**Figure 4:**
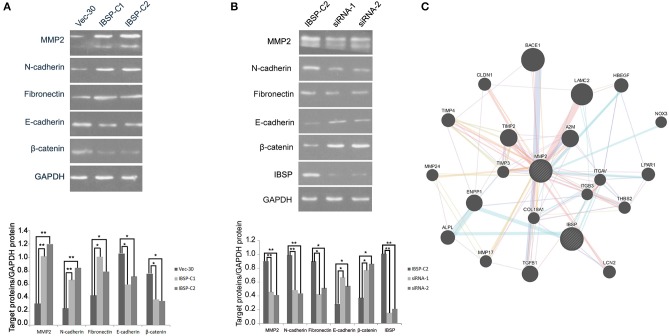
IBSP improves the invasion and metastasis ability of tumor cells by regulating the EMT process. **(A)** Western blot analysis was performed to compare the expression levels of epithelial and mesenchymal markers transfected with no load cells and IBSP cells. β-Actin was used as a loading control. **(B)** Western blot was used to detect the efficiency of IBSP gene silencing and the expression of various markers in IBSP highly expressed cells and siRNA interfered cells. β-Actin was used as a loading control. **(C)** The interaction network of IBSP proteins was analyzed through the GeneMANIA database, and MMP2 and IBSP were found to be closely related. ^*^P < 0.05, ^**^P < 0.01.

IBSP-C2, which expresses a high level of IBSP, was used in the siRNA experiment. siRNA targeting IBSP was tested and the efficiency of IBSP gene silencing was detected by western blot. The result showed that the expression of IBSP in the IBSP-C2 clone treated by siRNA was significantly decreased (Figure 4B).

### IBSP Regulates the EMT Process

To clarify the mechanism of IBSP in promoting tumor cell metastasis, the effect of IBSP on EMT-related epithelial markers (E-cadherin and β-catenin) and mesenchymal markers (N-cadherin and Fibronectin) was explored. As shown in Figure 4A, cell epithelial markers were downregulated in IBSP-transfected cells compared with their expression in the control group, while mesenchymal markers were upregulated. In addition, the expression of matrix metallopeptidase 2 (MMP2) in IBSP-transfected cells was significantly higher than that in the control group (*P* < 0.05). However, as can be seen from Figure 4B, compared with the IBSP-transfected cells, the expression of epithelial markers and MMP2 was up-regulated and the expression of interstitial markers was down-regulated in the IBSP cells treated with siRNA. In addition, as shown in Figure 4C, MMP2 and IBSP were closely related through the GeneMANIA database.

## Discussion

The IBSP gene is located on human chromosome 4q21.1 and encodes intracellular proteins containing 317 amino acids. IBSP is a member of the integrin-binding ligand N-linked glycoprotein (SIBLING) family, which also includes osteopontin, dental matrix protein, salivary phosphate protein, and extracellular phosphoglycoprotein (14, 22, 23). Studies have shown that although most members of the SIBLING family are mainly expressed in bone tissue, some proteins are abnormally expressed in malignant tumor tissue (14). The abnormal expression of the IBSP gene is closely related to bone metastasis, increased malignant risk and the poor prognosis of breast cancer, prostate cancer and non-small cell lung cancer.

In this study, we analyzed the differences in the expression of IBSP mRNA and protein in ESCC tissues and adjacent normal esophageal mucosa tissues by qRT-PCR and IHC. The results illustrated that both the expression of IBSP mRNA and protein in ESCC tissues was enhanced, indicating that the upregulation of the IBSP gene was closely related to the occurrence and development of ESCC. The mechanism of the increased expression of IBSP protein in malignant tumor cells is still unclear, but the relationship between the genes and clinicopathological features indicates that the upregulation of IBSP is more common in patients with lymph node metastasis (*P* = 0.017), advanced clinical stage (*P* = 0.001), and poor survival (*P* < 0.001). KM analysis indicated that the overall survival rate of ESCC patients decreased with the upregulation of IBSP in tumor tissues. Multivariate analysis showed that tumor differentiation degree, TNM stage and IBSP upregulation could be used as independent prognostic indicators for ESCC patients. In addition, we found that there was no significant difference in the survival rate between patients with IBSP upregulation and those without IBSP upregulation in pathological stage III. The possible reason is that the factors affecting the survival of patients with advanced tumors are relatively complex, such as lymph node metastasis, depth of tumor invasion and potential distant metastasis, which affected the overall survival. Therefore, expanding the sample size for further analysis is necessary.

Previous studies have revealed that integrins play an important role in tumor metastasis by regulating the invasion and migration of cells (24, 25). In addition, they can promote the adhesion ability of cells to the extracellular matrix and send and receive molecular signals regulating these processes. IBSP usually interacts with integrins of heterogeneous dimers on the cell surface, such as αvβ3 (26). IBSP acts on the integrin ligand, a highly conserved RGD sequence at the c-terminal of the proximal protein, to enhance the adhesion of various types of cells. A three-molecule complex consists of IBSP with αvβ3 and matrix metalloproteinase 2 (MMP2), which can enhance local matrix degradation and tumor cell invasion (27). In addition, IBSP interacts with complement factor H to form cell surface-related complexes that protect cells from complement-mediated lysis (28). As a binding molecule, IBSP plays an important role in the adhesion process between proteins and the surface of migrating cells. By stimulating the formation of molecular signals through adhesive plaques, it promotes the formation of the precursor of transfer factors (29).

The relation between IBSP and EMT was also illustrated in our study. EMT is a biological process in which tumor cells lose epithelial polarity and transform into the mesenchymal phenotype (30, 31). EMT endows cancer cells with metastatic properties that facilitate their invasion, migration, and subsequent spread, which plays an important role in malignant transformation (31). The enhancement and upregulation of N-cadherin function in epithelial cells or the weakening and downregulation of E-cadherin function are considered to be signs of EMT (32–34). In the present study, western blotting showed that mesenchymal markers (N-cadherin and Fibronectin) were upregulated in IBSP cells, while epithelial markers (E-cadherin and β-catenin) were significantly downregulated, suggesting that the effects of IBSP on ESCC progression might be partly associated with the EMT process. Furthermore, matrix metalloproteinases (MMPs) play a very important role in the EMT process and can improve the invasion and metastasis ability of tumor cells through the degradation of extracellular matrix components or regulation of cell signaling pathways (35). According to our study, IBSP can activate the MMP pathway by modulating the activity of MMP2 (36). The MMP2 gene is a member of the MMP gene family, which are zinc-dependent enzymes capable of cleaving components of the extracellular matrix and molecules involved in signal transduction (37, 38). As a member of the MMP superfamily, MMP2, existing on the surface of invading cells in the form of proteolytic activity, has the ability to hydrolyze basement membrane components. In summary, MMP2 is a key molecule in all aspects of tumor growth and metastasis (39–41). However, the exact role of IBSP in EMT progression remains unclear.

In summary, the results of this study showed that IBSP upregulation was often found in ESCC tumor specimens, indicating the poor prognosis of ESCC patients. Moreover, the results also demonstrated that IBSP could enhance the ability of ESCC cell proliferation and tumor metastasis, indicating that IBSP may be a valuable prognostic indicator in ESCC patients. Interference and knock out experiments will be performed in a later study to further confirm this conclusion. However, the mechanism by which IBSP promotes tumorigenesis and development needs more research.

## Data Availability Statement

All datasets generated for this study are included in the manuscript/supplementary files.

## Author Contributions

All authors listed have made a substantial, direct and intellectual contribution to the work, and approved it for publication.

### Conflict of Interest

The authors declare that the research was conducted in the absence of any commercial or financial relationships that could be construed as a potential conflict of interest.
